# Occlusal characteristics in 3-year-old children – results of a birth cohort study

**DOI:** 10.1186/s12903-015-0080-0

**Published:** 2015-08-07

**Authors:** Yvonne Wagner, Roswitha Heinrich-Weltzien

**Affiliations:** Department of Preventive Dentistry and Paediatric Dentistry, Jena University Hospital, Bachstr. 18, 07743 Jena, Germany

## Abstract

**Background:**

Aim of this prospective study was to determine prevalence of malocclusion and associated risk factors in 3-year-old Thuringian children.

**Methods:**

Subjects (*n* = 377) were participants in a regional oral health programme, a birth cohort study with the aim to prevent caries (German Clinical Trials Register DRKS00003438). Children received continuous dental care since birth. Occlusal characteristics (overjet, overbite, anterior open bite, canine relationship and posterior crossbite) were measured at the age of 3 years by one calibrated clinician using a vernier caliper (accuracy 0.1 mm; Münchner Modell 042-751-00, Germany). A regular parent survey was conducted to assess risk factors for development of malocclusion.

**Results:**

Three hundred seventy seven children (mean age 3.31 ± 0.70 years; 52.5 % male) were examined. Children had a mean overjet of 2.4 ± 0.8 mm and the mean overbite was 0.8 ± 1.2 mm; 58.8 % of the children had a normal overjet ≤3 mm and 88.8 % a normal overbite with < $$ {\scriptscriptstyle \raisebox{1ex}{$2$}\!\left/ \!\raisebox{-1ex}{$3$}\right.} $$ overlap. Prevalence of malocclusion was 45.2 % (10.9 % anterior open bite, 41.2 % increased overjet ≥3 mm, 40.8 % Class II/III canine relationship, 3.4 % posterior crossbite). All children who sucked the thumb had a malocclusion. Children who used a pacifier had greater odds of having a malocclusion at age of 3 years than children without pacifier use (OR = 3.36; 95 % CI: 1.87–6.05). Malocclusion and dental trauma were associated, but not statistically significant (OR = 1.83; 95 % CI: 0.99–3.34; *p* = 0.062). Malocclusion was not associated with gender, migration background, low socioeconomic status, preterm birth, special health care needs, breathing or dietary patterns (*p* > 0.05).

**Conclusions:**

Non-nutritive sucking habits were important risk factors for development of a malocclusion in the primary dentition.

## Background

Recently there has been an increased awareness of the role of the primary dentition in the development and occlusion of the permanent dentition [1–3]; for instance, discrepancies in the occlusal characteristics of the primary dentition could lead to similar occlusal problems in the permanent dentition [2–6]. Also, malocclusion in the primary dentition can have detrimental consequences on dento- and maxillofacial development and on the oral-health-related quality of life [5, 6].

The prevalence of malocclusion in the primary dentition varies between 20 and over 70 %, depending on the population studied and the criteria used for scoring [1, 5, 7, 8].

Malocclusion is a developmental condition that results from the interplay of genetic, environmental and various factors like the presence of oral habits [2–4, 9], such as the non-nutritive sucking on a thumb, digit, pacifier and other influences such as lips or tongue thrusting [10–12]. Non-nutritive sucking (NNS) is known to cause several changes in dental occlusion [2, 3, 10–19]. Children with pacifier- or digit-sucking habits are significantly more likely to develop anterior open bite, increased overjet, Class II canine relationship and posterior crossbite compared to children without a habit history [2, 3, 10–19]. The early diagnosis of the malocclusion and identification of aetiological factors is necessary for early management [1–5]. Studies have shown that when NNS habits are stopped, spontaneous resolution may occur [8, 16–19]; for instance, anterior open bite tends to resolve, although posterior crossbite and increased overjet persist after the cessation of the habit [18, 16–19]. The further physiological development and regular occlusion with correct dental arches and relations decrease the requirement for additional orthodontic treatment [2–6].

To date, studies have focused on the occlusal characteristics in the mixed and permanent dentition [1, 4, 8], while only a limited number of studies have assessed the occlusal characteristics in the primary dentition [4]. Furthermore, most of the studies have a cross-sectional design or are retrospective.

The primary aim of the present study was to determine the prevalence of malocclusion and associated risk factors in 3-year-old Thuringian children. The secondary aim was to specify standard occlusal characteristics for the primary dentition in children without any oral habits or prolonged feeding practice.

## Methods

### Study design

This study was part of a prospective cohort study to evaluate the impact of a preventive programme on the oral health of Thuringian children in Germany (German Clinical Trials Register DRKS00003438). The Ethics Committee of Jena University Hospital approved the study (registration number 2759-02/10). The study was conducted in full accordance with the ethical requirements of the World Medical Association Declaration of Helsinki (2008). This investigation complied with the recommendations of the STROBE statement guidelines.

### Study population

The study population included all the children from the Jena birth cohort 7/2009 to 10/2010 (*n* = 1162) who had participated in the final examination of the preventive programme (*n* = 377, 32.4 %). The eligibility criteria were the provision of written consent by the caregiver and the availability of data relating to the caregiver’s interview and dental examination of the child. The exclusion criteria were no written consent and incomplete data.

### Preventive programme

The participating children were included in a risk-related recall system with continuous oral care from birth up to the age of 3 years. A caries risk assessment was carried out using the Caries-risk Assessment Tool (CAT) for infants, children and adolescents of the American Academy of Pediatric Dentistry (AAPD) to categorise the children, who were also re-evaluated at each dental appointment [20]. Children with an increased caries risk were reappointed every 3 months and children with a low or moderate caries risk every 6 months. High-risk children received fluoride varnish application biannually.

### Dental examination

The children were examined at the Department of Preventive and Paediatric Dentistry, Jena University Hospital, Germany. The examinations were conducted using a dental light, mirror and a sterile gauze for removing debris and drying the teeth, and with the child sitting on their parent’s lap in an upright position in a dental chair so that the Frankfort horizontal plane was parallel to the floor. No radiographs were taken. Sagittal, vertical and transversal measurements were made with a vernier caliper (Münchner Modell 042-751-00, Germany) with an accuracy of 0.1 mm. Registration of the occlusal characteristics was carried out according to the principles developed by the Federation Dentaire Internationale [21]. The following occlusal parameters were recorded:

1. Amount of overjet in millimetres between two antagonistic anterior teeth (lateral or central incisor) measured from the facial surface of the most lingual mandibular tooth to the middle incisal edge of a more facially positioned maxillary tooth. An overjet ≥3 mm was considered to be an increased overjet.
2. Overbite measured in millimetres and recorded as the overlap of the mandibular anterior teeth by the maxillary anterior teeth. A pencil mark on the tooth indicating the extent of the overlap facilitated the measurement. The overbite was recorded as the amount of overbite in millimetres (mean overbite and standard deviation) as well as the degree of overbite, recorded as the percent overlap of the mandibular incisors crown (i.e. a one third covering of the lower incisors, between one third and two thirds covering and more than two thirds covering).
  The overbite was described as reduced when an anterior open bite was present. The amount of an open bite was measured directly in millimetres between the incisal edges of the maxillary and mandibular anterior teeth.
  The overbite was described as increased when more than two thirds of the lower incisors were covered.
3. Primary canine relationship, recorded as Class I, II or III.
4. Presence of a posterior crossbite, recorded as unilateral or bilateral. A posterior crossbite was considered when a reverse buccal overjet was present on one or both sides of the mouth.

In this study, malocclusion was defined as an incorrect occlusion or misalignment according to Angle [22] and included at least one of the following conditions: a Class II or III primary canine relationship, increased overjet (≥3 mm), anterior open bite and a unilateral or bilateral posterior crossbite.

All the measurements were performed by the same calibrated clinician (YW), who had been trained and calibrated following WHO guidelines by an experienced epidemiologist and who first practised the examination on a group of 10 subjects (RHW) [23]. Afterwards, the dentist examined a group of 25 pre-selected subjects twice on successive days to assess the consistency. Intra-rater reliability was good (Kappa = 0.80). Duplicate examinations were conducted at the beginning, after 1 year and at the end of the survey with 25 subjects included in the main survey. The intra-rater reliability was good (Kappa = 0.80 to 0.88).

### Questionnaire

The survey instrument was a standardised questionnaire, which was updated during each dental visit. The first complex included six questions about general information: age, gender of the child and special health care needs (general disease, preterm birth, syndromal diseases). The second complex contained ten questions about the feeding behaviour (bottle- and/or breastfeeding, use of a sippy cup, duration), NNS habits (thumb/digit sucking, use of a pacifier, duration, weaning time), breathing patterns, allergies and traumatic injuries.

The questionnaire was tested by two experts regarding validity and readability. Subsequently the revised questionnaire was tested and retested in a review group of 25 randomly selected parents before being used.

### Statistical analysis

Data were recorded in excel files and transferred to the Statistical Package for Social Sciences (SPSS version 20, IBM Corporation, Armonk, NY, USA). The data were analysed using the Fisher exact test to determine the statistical significant associations between the independent variables (feeding type, thumb sucking, use of a pacifier, etc.) and the outcome variable malocclusion. A p-value ≤0.05 was set to indicate statistically significant differences. Variables that showed significant associations were used as the basis for group allocation of the children. Group I formed the basis for the determination of norm occlusal characteristics in 3-year-old children. Children with NNS habits; prolonged feeding practices; congenital-, genetic- or trauma-caused misalignment; mouth breathing; dental caries; developmental defects of enamel or allergies were allocated to the other groups.

## Results

A total of 377 children (mean age 3.31 ± 0.70 years; 52.5 % male; dropout 26.4 %) with complete primary dentition were examined. Of these children, 45.2 % (*n* = 170) had a malocclusion. Descriptions of the independent variables of all the children and of the children with malocclusion are presented in Table 1. Statistically significant associations were found between malocclusion and pacifier use (*p* < 0.001) and thumb- or digit-sucking (*p* < 0.001). Children who used a pacifier revealed a 3.4 times higher probability (OR) of having a malocclusion at the age of 3 years than children without pacifier use (OR = 3.36; 95 % CI: 1.87–6.05). All the children who sucked their thumb or digit had a malocclusion at the age of 3 years compared to children without this habit. Malocclusion and dental trauma were associated, but not statistically significant (OR = 1.83; 95 % CI: 0.99–3.34; *p* = 0.062). Malocclusion was not associated with gender, migration background, low socioeconomic status, preterm birth, special health care needs, breathing or dietary patterns (*p* > 0.05) (Table 1). According to the statistical associations, children were assigned to one of four groups: Group I (*n* = 44) formed the basis for norm occlusion; Group II (*n* = 304) included children who used a pacifier; Group III (*n* = 16) were children who sucked their thumb or digit and Group IV (*n* = 13) included all the remaining children. The prevalence of malocclusion was 0.0 % in Group I, compared to 50.5 % in Group II, 100.0 % in Group III and 7.7 % in Group IV.

**Table 1:** Description of independent variables for all children and children with malocclusion

Variables	All children (*n* = 377)			Children with Malocclusion (*n* = 170)		Fisher exact test		
		%	(*n*)	%	(*n*)	OR	95 % CI	*P*-value
Gender	Male	52.4	197	47.2	93	0.84	0.56–1.27	0.468
	Female	47.6	180	43.0	77			
Migration background	Yes	6.1	23	39.1	9	0.77	0.32–1.82	0.667
	No	93.9	354	45.6	161			
Low socioeconomic status	Yes	9.3	35	40.0	14	0.79	0.39–1.61	0.594
	No	90.7	342	45.6	156			
Preterm birth	Yes	4.3	16	43.8	7	0.94	0.34–2.58	1.000
	No	95.7	361	45.3	163			
General disease/special health care needs	Yes	7.2	27	29.6	8	0.49	0.21–1.14	0.109
	No	92.8	350	46.4	162			
Mouthbreathing	Yes	0.5	2	100.0	2	^a^	^a^	0.204
	No	99.5	375	44.9	168			
Allergic rhinitis	Yes	1.6	6	33.3	2	0.60	0.11–3.32	0.694
	No	98.4	371	45.4	168			
Breastfeeding	Yes	66.8	251	44.2	111	0.89	0.58–1.36	0.660
	No	33.2	126	47.2	59			
Bottlefeeding >15 months	Yes	24.7	93	49.5	46	1.36	0.70–2.67	0.398
	No	75.3	284	41.8	23			
Drink learn cup	Yes	85.4	321	44.2	142	0.77	0.43–1.36	0.382
	No	14.6	55	50.9	28			
Pacifier	Yes	80.6	304	50.5	153	3.36	1.87–6.05	0.001
	No	19.4	73	23.3	17			
Thumb/finger sucking	Yes	4.3	16	100.0	16	^a^	^a^	0.001
	No	95.7	361	42.8	154			
Dental trauma	Yes	12.8	48	58.3	28	1.83	0.99–3.34	0.062
	No	87.2	329	43.3	142			

^a^All children with the independent variable had a malocclusion. Odds ratio (OR) was mathematically incalculable

Occlusal characteristics (overjet, increased overjet, overbite, anterior open bite, primary canine relationship, posterior crossbite) in the children of the different groups are presented in Table 2. Children had a mean overjet of 2.4 ± 0.8 mm, and the mean overbite was 0.8 ± 1.2 mm. Prevalence of the anterior open bite was 10.9 %, and 41.2 % of the children had an increased overjet. A Class I primary canine relationship was found in 59.2 % of the children, while Class II was found in 40.8 %. No child revealed the Class III relationship. The posterior crossbite was present in 3.4 % of the children. Children in Group I had a mean overbite of 1.4 ± 0.2 mm and a mean overjet of 1.2 ± 0.2 mm.

**Table 2:** Occlusal characteristics (overjet, increased overjet, overbite, anterior open bite, primary canine relationship, posterior crossbite) in all children according to group

	All children *n* = 377	Group I (norm) *n* = 44	Group II (pacifier) *n* = 304	Group III (thumb) *n* = 16	Group IV (other) *n* = 13
Overjet					
Mean overjet ± SD (mm)	2.4 ± 0.8	1.2 ± 0.2	2.6 ± 0.7	3.3 ± 0.4	1.3 ± 0.6
Increased overjet *N* (%)	155 (41.2)	0 (0.0)	138 (45.4)	16 (100.0)	1 (7.7)
Mean ± SD	3.2 ± 0.3	–	3.2 ± 0.3	3.3 ± 0.4	3.6 ± 0.0
Overbite					
Mean overbite ± SD (mm)	0.8 ± 1.2	1.4 ± 0.2	0.8 ± 1.2	−0.3 ± 2.1	1.3 ± 0.1
Anterior open bite *N* (%)	41 (10.9)	0 (0.0)	35 (11.5)	6 (37.5)	0 (0.0)
Mean ± SD	2.2 ± 1.4	–	3.4 ± 1.9	−2.6 ± 1.7	–
≤ $$ {\scriptscriptstyle \raisebox{1ex}{$1$}\!\left/ \!\raisebox{-1ex}{$3$}\right.} $$ overlap *N* (%)	212 (56.2)	21 (47.7)	174 (57.2)	10 (62.5)	7 (53.8)
$$ {\scriptscriptstyle \raisebox{1ex}{$1$}\!\left/ \!\raisebox{-1ex}{$3$}\right.} $$ to $$ {\scriptscriptstyle \raisebox{1ex}{$2$}\!\left/ \!\raisebox{-1ex}{$3$}\right.} $$ overlap *N* (%)	123 (32.6)	23 (52.3)	95 (31.3)	0 (0.0)	5 (38.5)
> $$ {\scriptscriptstyle \raisebox{1ex}{$2$}\!\left/ \!\raisebox{-1ex}{$3$}\right.} $$ overlap *N* (%)	1 (0.3)	0 (0.0)	0 (0.0)	0 (0.0)	1 (7.7)
Primary canine relationship					
Class I *N* (%)	223 (59.2)	44 (100.0)	162 (53.3)	5 (31.2)	12 (92.3)
Class II *N* (%)	154 (40.8)	0 (0.0)	142 (46.7)	11 (68.8)	1 (7.7)
Class III *N* (%)	0 (0.0)	0 (0.0)	0 (0.0)	0 (0.0)	0 (0.0)
Posterior crossbite					
Unilateral *N* (%)	13 (3.4)	0 (0.0)	12 (3.9)	1 (6.2)	0 (0.0)
Bilateral *N* (%)	–	0 (0.0)	0 (0.0)	0 (0.0)	0 (0.0)

## Discussion

This study showed that 54 % of the 3-year-olds had a malocclusion. The main results from the present study are that children who used a pacifier and children who sucked their thumb or digit had a higher probability of having a malocclusion at the age of 3 years than children without these habits. Malocclusion and dental trauma were also associated, but not statistically significant. Children without any NNS habits, a prolonged feeding practice or other influencing factors had no malocclusion or alteration in oral structures at the age of 3 years.

The present study is based on data from a regional German birth cohort study. Clinical and survey data were obtained on an ongoing basis and at regular intervals up to the age of 3 years. The results of this study are consistent with previous studies concerning the prevalence of malocclusions in the primary dentition and that showed that NNS habits were associated with an increased overjet and decreased overbite [10, 12, 16, 24, 25]. All the children in the thumb-sucking group and about 45 % of the children in the pacifier-sucking group exhibited an increased overjet of greater than 3 mm. Children with a thumb-sucking habit had a greater mean overjet (3.3 mm) and a lower mean overbite (−0.3 mm) than the pacifier-sucking children (mean overjet 3.2 mm; mean overbite 0.8 mm). Warren et al. [24] observed comparable findings (mean overjet 3.7 vs. 2.1 mm) [24]. Zimmer et al. [25] found an average overjet of 1.2 mm in 121 children aged 16 months who did not use a pacifier compared to 1.7 mm with those who used one. In the present study, children without any NNS habits had a mean overjet of 1.2 mm. Another remarkable finding was that all the children with a thumb-sucking habit had a malocclusion, whereas in the pacifier-sucking group the prevalence of malocclusion was about 50 %.

Studies suggest that an increased overjet and anterior open bite were predisposing factors of a dental trauma [26–28]. A protrusion of the maxillary incisors, an increased overjet of 3.5 mm or more, and inadequate lip coverage could be associated with an increased prevalence of dental trauma [26–28]. Young children start to crawl, stand, walk and run, and fall, such that dental injuries and dislocated teeth are common [29]. This study revealed no significant association between malocclusion and dental trauma, although a trend was observable, which might be strengthened in the permanent dentition. As older children start to take up high risk activities such as inline skating, hockey or martial arts, they are also predisposed to experience dental trauma [29].

An anterior open bite was found in 10.9 % of the total sample. Other studies showed a prevalence of the anterior open bite between 2.8 and 46.2 % [30–33].

The majority of the 3-year-olds in the present study revealed a Class I canine relationship; while, a Class II relationship was more frequent in children with an oral habit. These findings are comparable to other data, where a Class I canine relationship was observed in 57 % of the children, a Class II relationship in 29 % and a Class III relationship in 4 %, respectively [34]. A Class I canine relationship is more prevalent among children without widespread NNS [35].

The prevalence of posterior crossbite was present among 3.4 % of our 3-year-olds. Other studies reported a range from 7 to 17 % in the deciduous and early mixed dentition [26, 31, 33]. In relation to posterior crossbite, some studies found no significant difference between children with and without NNS habits, whereas other studies showed an association [5, 24, 26, 31, 33].

Some limitations of this study need to be addressed. First, this study was limited to a relatively small geographic location and to those children who participated in the preventive programme. Consequently, the findings are restricted to this population group. To reduce the source of potential bias, all the data of the children were recorded longitudinally, starting at the time of birth up to the age of 3 years. Another limitation of the study pertains to the intra-oral measurements in children aged 3 years due to their stage of development. To ensure comparable measurements and to stabilise the child, a parent sat in the dental chair with the child in their lap, though all the measurements were re-checked for accuracy.

The present study found a significant relationship between thumb- or pacifier-sucking and the development of malocclusion. The association between malocclusion and dental trauma was not significant. It could be shown that children without any oral habits had an ideal occlusion. Therefore, at the age of 3 years, the occlusal development should be observed and the parents should be informed about the possible undesirable consequences of prolonged NNS habits. It is planned to follow-up with the children into the age of the mixed and permanent dentition to record the development of the occlusal characteristics, and to determine the frequency of spontaneous correction.

## Conclusion

NNS habits are important risk factors for the development of malocclusion in the primary dentition.

## Abbreviations

AAPD: American Academy of Paediatric Dentistry
NNS: Non-nutritive sucking
STROBE: Strengthening the reporting of observational studies in epidemiology
